# New insights on testicular cancer prevalence with novel diagnostic biomarkers and therapeutic approaches

**DOI:** 10.1002/cnr2.2052

**Published:** 2024-03-20

**Authors:** Kadry M. Sadek, Hazem Y. AbdEllatief, Sahar F. E. Mahmoud, Athanasios Alexiou, Marios Papadakis, Marwan Al‐Hajeili, Hebatallah M. Saad, Gaber El‐Saber Batiha

**Affiliations:** ^1^ Department of Biochemistry, Faculty of Veterinary Medicine Damanhour University Abadiyyat Damanhur Egypt; ^2^ Department of Histology, Faculty of Veterinary Medicine Damanhour University Abadiyyat Damanhur Egypt; ^3^ University Centre for Research and Development Chandigarh University Mohali Punjab India; ^4^ Department of Research and Development, Funogen Athens Greece; ^5^ Department of Research and Development AFNP Med Wien Austria; ^6^ Department of Science and Engineering Novel Global Community Educational Foundation Hebersham New South Wales Australia; ^7^ Department of Surgery II University Hospital Witten‐Herdecke Wuppertal Germany; ^8^ Department of Medicine King Abdulaziz University Jeddah Kingdom of Saudi Arabia; ^9^ Department of Pathology, Faculty of Veterinary Medicine Matrouh University Marsa Matruh Egypt; ^10^ Department of Pharmacology and Therapeutics, Faculty of Veterinary Medicine Damanhour University Damanhour Egypt

**Keywords:** cancer, diagnosis, germ cell, male, seminoma, testis, treatment

## Abstract

**Background:**

Testicular cancer (TC), comprising merely 1% of male neoplasms, holds the distinction of being the most commonly encountered neoplasm among young males.

**Recent findings:**

Most cases of testicular neoplasms can be classified into two main groups, namely germ cell tumors representing approximately 95% of the cases, and sex cord‐stromal tumors accounting for about 5% of the cases. Moreover, its prevalence is on the rise across the globe. TC is a neoplastic condition characterized by a favorable prognosis. The advent of cisplatin‐based chemotherapeutic agents in the latter part of the 1970s has led to a significant enhancement in the 5‐year survival rate, which presently surpasses 95%. Given that TC is commonly detected before reaching the age of 40, it can be anticipated that these individuals will enjoy an additional 40–50 years of life following successful treatment. The potential causes of TC are multifactorial and related to different pathologies. Accurate identification is imperative to guarantee the utmost efficacious and suitable therapy. To a certain degree, this can be accomplished through the utilization of blood examinations for neoplastic indicators; nonetheless, an unequivocal diagnosis necessitates an evaluation of the histological composition of a specimen via a pathologist.

**Conclusion:**

TC is multifactorial and has various pathologies, therefore this review aimed to revise the prenatal and postnatal causes as well as novel diagnostic biomarkers and the therapeutic strategies of TC.

## 1 INTRODUCTION

Testicular cancer (TC), comprising merely 1% of male neoplasms, holds the distinction of being the most commonly encountered neoplasm among young males. Moreover, its prevalence is on the rise across the globe.^1^ TC is the most common solid malignant tumor at a young age.^2^ In the last years, cases of TC have increased and become more important because of the long‐term effect of both the disease and its treatment throughout a patient's lifetime.^3^ Understanding the origin of cell cancer and identifying its specific type are of utmost importance due to the variations in treatment strategies and prognostic outcomes.^4^, ^5^ TC denotes a neoplasm that exhibits favorable outcomes with high cure rates.^4^, ^5^ Since the advent of cisplatin‐derived chemotherapeutic agents in the latter part of the 1970s, there has been a significant augmentation in the 5‐year survival rate, which presently surpasses the threshold of 95%.^6^ As TC is commonly identified prior to reaching the age of 40, these individuals may anticipate an additional lifespan of 40–50 years after receiving effective medical intervention.^7^ This achievement, nevertheless, is impeded by an elevated vulnerability to enduring and delayed repercussions of therapy. Subsequent malignant neoplasms and cardiovascular ailment symbolize the most prevalent potentially life‐threatening belated repercussions, typically manifesting more than a decade following therapy.^6^, ^7^ Other enduring consequences comprise pulmonary, kidney and nervous toxicities, reduced fertility, hypogonadism, and psychosocial problems.^6^, ^7^ The frequency and duration of these different unfavorable outcomes differ depending on the type of therapy and its level of severity. There is insufficient knowledge regarding the underlying mechanisms and genetic predisposition to the manifold detrimental consequences. Apart from the burden of treatment, it remains challenging to ascertain individuals who are highly susceptible to specific long‐term consequences following TC intervention.^6^, ^7^ A common early manifestation of TC is frequently the presence of a mass or enlargement in the testes. The recommendation of the United States Preventive Services Task Force (USPSTF) is to discourage the regular screening for TC among asymptomatic adolescents and adults, which includes the routine practice of testicular self‐examinations.^6^ The American Cancer Society proposes that periodic testicular examinations be conducted by certain individuals of the male population, particularly those with a familial predisposition to cancer. Similarly, the American Urological Association advocates for the regular self‐examination of the testes every month, extending this recommendation to all young males.^8^ Symptoms may potentially encompass the presence of one or more of the subsequent manifestations: an anomalous mass located within one of the testes, which could potentially be accompanied by varying degrees of discomfort, a piercing sensation or a muted twinge in the inferior region of the abdominal cavity or scrotum, a sensation frequently characterized as “heaviness” within the scrotal region, and an increased rigidity of the testicular structure.^9^ Moreover, the hormonal effects of the beta‐subunit of human chorionic gonadotropin (hCG) can lead to breast enlargement, also known as gynecomastia^10^ and the occurrence of lumbago, also known as low back pain, is attributed to the dissemination of cancer cells to the lymph nodes situated along the spinal region.^5^, ^11^ It is infrequent for TC to metastasize to other organs, except for the pulmonary system.^5^, ^11^ If it possesses, nevertheless, the subsequent indications may potentially be evident: an insufficiency of breath (dyspnea), expulsion of mucus or the expectoration of blood (hemoptysis) resulting from the dissemination of cancer to the pulmonary region, and a lumps in the cervical region due to the metastasis of cancer to the lymphatic nodes.^5^, ^11^ Testicular dysgenesis syndrome encompasses the constituents of TC, cryptorchidism, hypospadias, and suboptimal semen quality.^5^, ^11^

The potential causes of TC are multifactorial and related to different pathologies. Cryptorchidism, commonly known as undescended testicles, is a significant risk factor contributing to the onset of TC.^12^ The prevailing belief is that the existence of cryptorchidism plays a role in the development of a tumor; in cases where cryptorchidism coexists with a tumor, the tumor tends to be of considerable size.^12^ Other potential risk factors comprise inguinal hernias and Klinefelter syndrome,^12^ and mumps orchitis.^13^ Physical activity is linked to a reduced risk, while a sedentary way of life is correlated with an elevated risk of TC.^14^ Increased risk is linked to the occurrence of male characteristics at an early stage.^15^ This could potentially arise from endogenous or environmental hormonal influences. The incidence of testicular cancer appears to be elevated in Western countries, which has been attributed to the consumption of cannabis.^16^

Based on the histopathological classifications of testicular cancer, it is noteworthy that while testicular cancer can originate from any specific cell type present in the testicles, the overwhelming majority, accounting for more than 95% of cases, are attributed to germ cell tumors (GCTs).^17^ A majority of the remaining 5% comprises neoplasms known as sex cord‐gonadal stromal tumors, which originate from either Leydig cells or Sertoli cells.^4^, ^5^, ^17^ GCTs constitute the most prevalent histological type.^18^ GCTs are categorized as seminoma, non‐seminoma as (teratoma) and mixed tumors.^18^, ^19^ Non‐seminomatous tumors are much more prevalent in the third decade, but pure seminoma is much more prevalent in the fourth. Early diagnosis, accurate staging, and a multidisciplinary therapeutic strategy can all lead to a favorable prognosis.^18^, ^19^

Accurate determination is imperative to guarantee the utmost efficient and suitable intervention.^20^ To a certain degree, this task can be accomplished through the utilization of blood examinations targeting specific markers indicative of neoplastic growth. However, a conclusive determination mandates the meticulous scrutiny of the histopathological composition of a sample, a task entrusted to a specialist pathologist.^2^, ^20^ TCs are most effectively categorized through the performance of radical inguinal orchiectomy. This surgical procedure enables the comprehensive histologic assessment of the entire testicle while also ensuring localized tumor management.^2^, ^20^

### 1.1 Epidemiology

Although TCs are uncommon, GCTs are the most frequent in young people. Ninety‐five percent of TC in post‐pubertal men are produced by germ cells, and the majority of cases affect individuals between the ages of 20 and 35.^21^ TC is common in Western and Northern Europe (8.7 and 7.2 per 100 000 males, respectively), with Western Asia having the greatest fatality rates. Most nations with low death rates attribute this to early identification by self‐examination and multi‐model therapy.^22^ In industrialized countries, the incidence has steadily increased during the last few decades. The bulk of these tumors are formed from germ cells (seminoma and nonseminoma germ cell testicular cancer), and more than 70% of patients are identified with stage I disease. Currently, testicular cancers have high cure rates, owing to early detection and their great chemo‐ and radiosensitivity.^23^ In the United States, testicular tumor is found among men between the ages of 20–34 years (51% of total cases). 22.9% of the cases are found in the ages of 35–44 years, 12.9% in the ages between 45 and 54 years, and the rest in other ages. The main age of diagnosis is 33 years.^24^ TC is highly correlated with ethnicity, as evidenced by the significant disparity in incidence rates between white and black individuals in the United States and southern England.^17^

These observations illustrated that TC is multifactorial and has various pathologies, therefore this review aims to revise the prenatal and postnatal causes as well as therapeutic strategies of TC.

### 1.2 Etiology and causes of TC

The etiology of TC has not been identified. The term “rising occurrence” refers to the impact of environmental factors on the incidence of additional testicular disorders such as sperm count reduction and increased testicular disease incidence.^21^ It has been postulated that the significant rise in prevalence can be attributed to certain environmental exposures that are becoming more widespread. These exposures are believed to have an early impact on life, thereby accounting for the birth cohort effects and the peak in prevalence during early adulthood. At the end of the 1970s, it has been shown that pregnancy hormone levels during fetal life play a significant role in TC.^25^ Since the early 1980s, the primary emphasis of etiological investigation has been directed towards prenatal and perinatal elements.^26^ Histopathological examinations have provided evidence indicating that the cellular composition of testicular cancer in situ (CIS) bears a striking resemblance to primordial germ cells and gonocytes.^27^ Based on these and various other observations, it has been postulated that factors encountered during the prenatal period disrupt the typical maturation of the rudimentary germ cells or gonocytes, resulting in the formation of CIS, which ultimately transforms into discernible malignancies in the adult stage.^26^, ^27^ Recently, it has been suggested that the identical though unidentified exposures may be responsible for inducing subfertility, cryptorchidism, and hypospadias. These conditions, in conjunction with testicular cancer, have been classified under the term Testicular Dysgenesis Syndrome (TDS).^28^

### 1.3 Inherited susceptibility for TC

Familial studies present the most substantial support for the presence of inherited susceptibility.^29^, ^30^ According to the data obtained from databases focused on cancer within families, it has been observed that male siblings of individuals diagnosed with TC exhibit a significantly elevated eight‐fold risk of developing the same condition. Moreover, the male members of the family, such as fathers and sons, display a four‐fold increase in their susceptibility to acquiring TC.^29^ Although the risks within a family can arise from both genetic factors and shared environmental influences, it has been approximated that the impact of environmental exposure, which is potent enough to independently generate the observed familial trends, would necessitate complete concurrence among siblings and elevate the risk by over 10 times.^31^ An analysis of segregation conducted using data from Norway and Sweden has indicated that there is a recessive pattern of inheritance, with a penetrance rate of 43% among individuals who are homozygous.^32^ As TC is relatively rare, no twin study with an adequate sample size has been conducted so far.^33^

An International Testicular Cancer Consortium was established in the early 1990s to conduct a linkage study on families with at least two affected cases of TC due to the evidence of an effect of inherited susceptibility. However, the most recent update does not provide any definitive evidence of a connection to any locus,^34^ probably indicating that similar to numerous other malignancies and persistent ailments, there exist multiple genes with moderate risk involved.^35^ Association studies are arguably the most effective methodology for ascertaining the identities of those genes. However, there are currently no suitable candidates and exploratory investigations are required within the context of very large research.^35^, ^36^ Holzik and colleagues' association studies have first focused on the role of HLA genes, with inconsistent results.^37^ As well, studies have investigated variants in genes involved in modulating the action of sex hormones. For example, no association has been found between the androgen receptor gene and TC risk in two case–control studies including about 100 cases each.^38^, ^39^ A study conducted in the United States examined multiple genes responsible for encoding enzymes involved in the metabolism of estrogen, assessing the variations in both the mother and the index subject. The study was based on the assumption that significant exposures would occur during the fetal stage.^40^ Investigations have been conducted on microdeletions in the Y chromosome in relation to the risk of decreased fertility and TC.^41^ Most of the limited number of studies conducted thus far, which were not subject to control, have yielded unfavorable outcomes.^37^ However, a collective investigation revealed an elevated susceptibility to TC linked to the removal of a singular portion of the Y chromosome.^41^ Although the study included only 2.3% of TC cases with the deletion, these findings are particularly noteworthy due to the association of the ‘gr/gr’ deletion with subfertility and its potential transmission from father to son.^42^, ^43^

Overall, there exists substantial evidence pertaining to a hereditary predisposition to TC, mediated through multiple genes of moderate risk. Although it is unlikely that a small number of TC cases can be solely attributed to hereditary susceptibility, the identification of the intricate genes and a deeper understanding of the underlying mechanisms behind these associations could greatly contribute to the comprehension of TC's etiology.

### 1.4 Prenatal and perinatal factors

Studies have been conducted to differentiate the impact of genetic susceptibility and environmental exposures to ascertain the risk of TC among migrants.^44^, ^45^ Some studies on migration have been successful in examining the occurrence of TC in both the initial and subsequent generations.^44^, ^45^ A multitude of these investigations have been conducted in the Nordic nations, capitalizing on the realization that Finland exhibits a low frequency, Sweden demonstrates a moderate frequency, and Denmark exemplifies a high frequency as a nation.^46^ Finnish males who have migrated to Sweden have consistently sustained their comparatively diminished susceptibility to risk, regardless of the age at which their migration took place. Conversely, the likelihood of testicular cancer in their offspring tended to converge with that of Swedish males, albeit it is worth noting that the latter investigation was conducted with a mere six instances.^47^, ^48^ Similarly, Danish males who relocate to Sweden have upheld their elevated susceptibility. In the subsequent generation, the vulnerability remained elevated if both parents were of Danish descent, whereas it corresponded with the average populace of Sweden if only one parent possessed Danish heritage.^49^ Hence, these investigations do not unequivocally eliminate the possibility of a hereditary predisposition, yet they consistently suggest that environmental elements exerting their influence during the early stages of existence play a pivotal role in the development of TC.

Numerous investigations have explored the correlation between various ‘indicators’ of prenatal exposures and the risk of testicular cancer, encompassing birth order, length of gestation, maternal age, presence of nausea during pregnancy, presence of bleeding during pregnancy, birth weight, and the occurrence of neonatal jaundice.^50^, ^51^, ^52^, ^53^ A comprehensive analysis of studies examining the correlation between low birth weight and the risk of developing TC revealed that the investigation of low birth weight has been undertaken in numerous studies primarily as an indicator of the impairment of fetal growth.^54^

An association between the act of smoking by mothers and the occurrence of TC has been postulated,^55^ while some case–control studies that relied on questionnaires have failed to find any correlation between maternal smoking and the risk of testicular cancer.^56^ Since the evaluation of the exposure was conducted in a retrospective manner, it is plausible that these studies could yield inaccurately pessimistic results. However, a study employing a case–control design and utilizing a prospective measurement of maternal smoking was conducted. This study examined a total of 192 Swedish men diagnosed with testicular cancer and 494 individuals serving as controls. The results of this study did not reveal any significant association between maternal smoking and TC.^57^

Additionally, there have been suggestions that the exposure of the fetus to hormones, specifically diethylstilbestrol (DES)—a non‐steroidal estrogen that has historically been utilized to inhibit abortions and alleviate pregnancy complications—is closely correlated with the likelihood of developing vaginal adenocarcinoma, thus leading to the potential occurrence of TC.^58^ The utilization of the pharmacological substance terminated in the year 1971, and individuals born before this era have surpassed the chronological stage of their lifespan during which testicular germ cell tumors are typically identified. Although it is highly unlikely that the rise in TC incidence over time can be attributed to prenatal exposure to DES, there are widespread suspicions surrounding other exposures to xenoestrogens and their potential impact on the incidence of GCT. The task of designing studies to furnish corroborative evidence of this phenomenon will undoubtedly present challenges. It is hoped that such studies can be aptly devised, leading to a more profound understanding of the fundamental cause for the observed rise in TC rates during the previous 40‐year period.^58^ A comprehensive examination and synthesis of multiple studies determined that occurrences during pregnancy, such as encountering substances possessing characteristics similar to those of DES, could potentially play a role in the development of testicular cancer.^59^ There is no discernible augmentation in the rates of either general or prostate cancer in men who were exposed to DES prenatally when compared to individuals who were not exposed to DES.^59^ A fortuitous mitigation of risk was noted in regards to cancers of the urinary system in individuals who were exposed, as compared to those who were unexposed. These discoveries imply that exposure to DES during the prenatal period is improbable to have a significant impact on the development of cancer in men of middle age.^60^

Additionally, there were higher levels of certain endocrine‐disrupting chemicals such as chlorinated biphenyls (PCBs) and hexachlorobenzene (HCB) detected in the blood of 44 mothers of TC patients compared to the blood of 45 mothers in the control group.^61^ The present study was subject to several limitations, notably the small sample size,^62^ the hypothesis holds substantial merit and justifies further investigation. A study utilizing multivariate logistic regression on a sample size of 103 cases and 215 controls revealed a correlation between the presence of seminoma and non‐seminoma and engaging in hobby activities that may have involved the utilization of endocrine‐disrupting compounds (EDCs) (such as paints, adhesives, or solvents). During the period of adolescence, living in rural areas exhibited a correlation with all variations of histological types of testicular cancer, including seminoma. No significant correlation was identified between the subjects' occupational exposures and the occurrence of this disease. The findings of this study additionally substantiate risk factors that have been previously identified, including a background of cryptorchidism and undescended testicles, a high level of education, and a low birth weight. Furthermore, it provides some backing for the hypothesis of a potential link between exposures to EDCs and the development of TC.^63^

On the whole, nevertheless, drawing a definitive conclusion from these studies presents a challenge due to the overall lack of consistency in the findings. It is conceivable that certain perinatal indicators might be influenced more by genetic factors rather than environmental exposures. Furthermore, it is still necessary to ascertain the specific environmental exposure that the perinatal ‘indicators’ are measuring.

### 1.5 Postnatal factors

Despite the existence of multiple research findings indicating the in‐utero origin of TC, the possibility cannot be dismissed that certain events occurring later in childhood or adolescence may trigger or facilitate the progress of germ‐cell cancer. Some attributes that occur after birth have consistently exhibited a correlation with a heightened likelihood of developing TC.^64^ Specifically, the majority of studies in which they have been investigated have reported early puberty and subfertility as risk factors.^64^ Regarding perinatal factors, the underlying mechanisms responsible for these associations remain undisclosed.

One particular temporal interval during which exposure to environmental elements may hold significant significance is the stage of puberty, characterized by heightened replication activities, such as that of spermatogonia. This particular cell type, which exhibits limited activity during infancy and childhood, commences the process of meiosis upon entering puberty, leading to the formation of spermatocytes and spermatozoa. This initiation is instigated by the surge of both steroid and peptide hormones.^65^ A phenomenon observed in a specific birth cohort, such as the one exemplified by TC, can be easily reconciled with a critical period that is dependent on age, such as puberty.^65^ The distribution of age in TC permits the occurrence of exposures to take effect before reaching the age of 18.^66^, ^67^

It would be of interest to conduct research specifically focused on examining the effects of exposures occurring during the period of development known as puberty. For example, a study based on correlational findings has suggested that consumption of milk may serve as a potential risk factor,^68^ and several of the case–control studies that have examined dietary factors have observed a correlation between the intake of dairy products and the likelihood of developing TC.^69^, ^70^ An investigation examining exposures during the stage of puberty should ideally assess the intake of milk during this specific timeframe and, if feasible, substantiate this data by means such as conducting interviews with the mothers.^70^ Additionally, it has been recognized that transition series metals such as cadmium, mercury, and cobalt possess carcinogenic properties based on numerous experimental investigations conducted on both animal and cell models.^70^

### 1.6 The link between cryptorchidism and TC

The failure in the descent of the testicles, also known as cryptorchidism, represents the prevailing anomaly observed in newborn males.^62^ The movement of the testes in the course of development is regulated by a peptide called insulin‐like 3 and steroid hormones that are manufactured in the Leydig cells of the testes. Additionally, various factors related to genetics and development also play a role in this phenomenon.^62^ In certain instances, although there have been demonstrations of a link between genetic abnormalities and environmental influences, the precise cause of cryptorchidism remains indeterminate. The frequency of cryptorchidism exhibits some degree of variation across different nations, with the most notable occurrence being observed in Denmark (9.0%), in contrast to the lowest recorded in Finland (2.4%).^71^ The accepted global prevalence rates of cryptorchidism generally range from 2% to 4%. The ingestion of smoked items, which have been associated with elevated occurrences of cryptorchidism, is a contributing factor.^72^ Similarly, the ingestion of more than five alcoholic drinks per week by mothers has been associated with an elevated risk of developing cryptorchidism.^72^

Cryptorchidism is a well‐established determinant of male infertility and TC.^12^ Experimental animal models indicate that an anomalous positioning of the testes can contribute to the disruption of spermatogenesis. Nonetheless, the association between cryptorchidism and TC is not as straightforward. Seminoma, which is widely regarded as the prevailing histological type of TC found in cryptorchid testes, is postulated to have its origins in pluripotent germ cells during prenatal development.^73^ Seminoma, which is widely regarded as the prevailing histological type of TC found in cryptorchid testes, is postulated to have its origins in pluripotent germ cells during prenatal development.^73^, ^74^ Approximately 10% of all instances of testicular germ cell tumors (TGCT) manifest in males who have a past medical record of cryptorchidism.^75^ Additionally, the condition of cryptorchidism exhibits a correlation with a heightened risk of TC, ranging from two to eightfold, for the development of TC.^75^ The association, while established, still leaves uncertainty regarding the biological mechanism that underlies it. This uncertainty arises from the possibility that TC and cryptorchidism might share environmental and/or genetic risk factors, or that it is the ectopic position itself that serves as a postnatal risk factor for TC. Moreover, the risk of cryptorchidism was found to be heightened in the contralateral testis, albeit not to the same degree as observed in the ipsilateral testis. This suggests the involvement of both shared prenatal risk factors and the postnatal positioning of the testes in contributing to the development of this condition.^76^ Several studies have examined whether the age at which orchiopexy is performed influences the likelihood of developing testicular cancer. This is based on the belief that if cryptorchidism is merely an indication of prenatal risk factors or genetic predisposition to testicular cancer, then surgical intervention would have no impact on altering the risk.^77^ Most of the studies conducted on this topic, which had a small sample size, discovered that there is an elevated risk of testicular cancer among individuals who undergo surgery at later stages of life. However, it is highly probable that the comparison between patients who received treatment at a later stage and those who were treated early is biased. This bias arises from the fact that the latter group consists of a certain proportion of boys who may have undergone unnecessary treatment for conditions such as retractile testis. These conditions typically resolve spontaneously and have a weaker or no association with testicular cancer.^53^, ^78^ Hence, a potential disturbance in a prevalent regulatory pathway, such as the androgen signaling mechanism, could potentially serve as an underlying causative factor for the correlation between cryptorchidism and TC.^79^, ^80^ An alternative hypothesis posits that the anomalous positioning of the testis is the primary factor contributing to both infertility and the development of germ cell tumors.^81^ Indeed, various studies have provided evidence for the causal relationship between an anomalous positioning of the testes and the occurrence of infertility.^82^, ^83^ The increased temperature of the testis that has not descended hampers the process of spermatogonia differentiation, leading to a halt in spermatogenesis, a decrease in the size of seminiferous tubules, depletion of germ cells, and the development of fibrosis.^84^ The association connecting cryptorchidism and TC, while not as well‐defined, remains somewhat ambiguous. Among cases of cryptorchidism, seminoma is the prevailing type of testicular germ cell tumor. The anomalous positioning of the testis significantly modifies the performance of somatic cells, creating an environment conducive to the self‐renewal and differentiation of spermatogonial stem cells.^85^ Moreover, the blood‐testis‐barrier (BTB), which is controlled by Sertoli cells, can be disrupted. This disruption has been observed in cryptorchid mice due to the breakdown of inter‐Sertoli cell tight junctions. This breakdown can potentially affect the development of later‐stage germ cells. Recent findings indicate that the cystic fibrosis transmembrane conductance regulator (CFTR), which plays a role in enhancing Sertoli cell tight junctions, is significantly downregulated in cryptorchid testes. This downregulation may contribute to the disruption of the BTB.^86^ This hypothesis was further substantiated by the revelation that subjecting primary Sertoli cell cultures to an incubation temperature of 37°C led to a notable reduction in CFTR expression when compared to those cultured at 32°C. The physiological division between germ cells before and after meiosis, which is upheld by Sertoli cells in the testis, guarantees that regulatory substances are directed towards specific groups of germ cells. Interference with their cellular structure, the BTB, and the expression of growth factors might result in the mismanagement of germ cells and anomalous differentiation, potentially culminating in the emergence of TC.^86^

Hence, it is our ultimate inference that preceding investigations corroborate the preventive impact of prompt surgical intervention, albeit a more extensive collection of data is mandatory to furnish decisive outcomes about this matter. The mechanism behind the correlation linking cryptorchidism and TC remains inexplicable, however, it is postulated that this could potentially be attributed to both shared etiology as well as the ectopic location of the testis, thereby conferring a predisposition to TC per se.

### 1.7 The link between subfertility and TC

It has been established that individuals diagnosed with TC exhibit compromised fertility during the period of cancer detection.^87^ Nevertheless, with regard to cryptorchidism, there exists a contentious discussion surrounding the mechanism that underlies the correlation. The association could potentially be elucidated by shared susceptibility, shared risk factors during both the prenatal and postnatal periods, or even the concept of reverse causality.^87^ Measuring subfertility presents a challenging endeavor, while the endeavor of devising studies to differentiate among these three mechanisms, which are not mutually exclusive, poses an even greater difficulty. A study was conducted to examine the correlation between the number of siblings and the occurrence of TC in a case–control study involving approximately 3000 cases and 9000 controls. The results revealed that there exists an inverse relationship between sibship size and the likelihood of developing TC.^46^ The aforementioned correlation experienced a decline as time progressed and ultimately ceased to exist within the population of individuals born subsequent to 1960.^46^ The potential link between birth order and the risk of developing TC has been proposed. Furthermore, it has been observed that sibship size has a significant correlation with birth order. Moreover, sibship size can serve as an indicator of parental fertility. Additionally, the diminishing strength of this association over time can be attributed to the decreasing significance of the number of children as a proxy for fertility.^46^ The siblings of individuals afflicted with testicular cancer displayed a tendency towards siring a diminished quantity of offspring, as well as a reduced proportion of twins of the opposite sex. This correlation, while only marginally significant from a statistical standpoint, was observed for both aforementioned markers.^88^ Multiple cohort studies have observed a correlation between infertility and heightened susceptibility to both testicular and prostate cancer.^89^, ^90^ There is a growing body of evidence indicating that there may exist various mechanisms that could potentially account for the heightened susceptibility to cancer in males experiencing subfertility. Y‐chromosome deletions, epigenetic hypermethylation, deletions/mutations of DNA mismatch repair genes, and aneuploidy are all mechanisms that have been observed in cellular cultures, animal models, and human tissues. The permeation of the interstitial space with an injected tracer dye demonstrated the disruption of the BTB in both scenarios.^86^ These mechanisms have been demonstrated to play a role in both infertility and cancer.^91^ TC exhibits a strong correlation with atypical semen parameters across all metrics.^92^ The administration asserts that information has additionally been utilized to recognize males with reduced fertility when biological specimen information is not accessible. Eisenberg et al.^93^ provided that utilizing the Current Procedural Terminology codes as a means of diagnosing male infertility led to an elevated likelihood of being diagnosed with testicular cancer, as indicated by hazard ratios of 1.49 and 1.78. A potential link has been proposed between the global decline in male reproductive capacity and the occurrence of TC,^94^ although the evidence regarding the existence of this particular type of association has yielded inconsistent results, the precise cause of such a correlation continues to be a subject of debate. A plausible biological connection can be established between subfertility and an elevated risk of testicular cancer. Firstly, it is crucial to consider the significance of fetal and neonatal stages in the onset of reproductive disorders. Several investigations have demonstrated that being exposed to an excessive amount of environmental estrogens (EES) during intrauterine life constitutes a risk factor for both subfertility and testicular cancer.^95^ Though the precise mechanism through which these effects occur remains poorly comprehended, certain researchers posit that EES not only hampers the ordinary endocrine functioning of the testis in a direct manner, but also perturbs the hypothalamic–pituitary‐testis axis, consequently resulting in the aberrant performance of Sertoli cells. This disruption ultimately impairs the differentiation of germ cells, subsequently leading to their transformation and eventual development into carcinoma in situ (CIS).^96^ Estimations anticipate that CIS will progress into the manifestation of testicular cancer within a span of 5 years subsequent to diagnosis, manifesting in 50% of all instances, whereas every patient harboring CIS cells during puberty will inevitably develop TC.^96^ Additionally, there is compelling evidence from genetic abnormalities that supports the existence of a correlation between subfertility and an elevated susceptibility to testicular cancer. Siblings of individuals with testicular cancer display reduced fertility rates and an increased likelihood of developing testicular cancer compared to controls of the same age.^97^

These findings suggest that there may be an increased prevalence of shared genetic predisposition among individuals with a confirmed background of reduced fertility in cases of TC.

### 1.8 Biomarkers of TC

Research efforts are currently underway to ascertain distinctive biomarkers of TC that possess the potential to enhance the process of diagnosing the disease, monitoring tumor development or recurrence, and evaluating the efficacy of therapeutic interventions. In the realm of diagnosing, predicting outcomes, and conducting surveillance for TC, three serum biomarkers have emerged as considerably reliable indicators that exhibit both specificity and sensitivity.^98^ These serum markers as α‐fetoprotein (AFP), human chorionic gonadotropin (HCG), and lactase dehydrogenase (LDH).^98^, ^99^ Individuals who have increased levels of serum LDH, AFP, or HCG, as well as their levels before receiving chemotherapy, have been incorporated in the prognosis of non‐seminomatous classification by the International Germ Cell Cancer Consensus Group (IGCCCG).^98^, ^99^ These patients are categorized into prognostic groups of good, intermediate, and poor based on the location of the primary tumor, levels of serum tumor markers, and the presence of extrapulmonary visceral metastases.^98^, ^99^ AFP, a glycoprotein with a molecular weight of 70 kD, is synthesized by the fetal yolk sac, liver, and gastrointestinal tract. Elevated concentrations of AFP are commonly observed in non‐seminomatous tumors such as embryonal carcinoma and yolk sac. The estimated half‐life of AFP is approximately 5–7 days.^100^ AFP levels are generally not raised in seminomas; however, in cases where elevated levels of AFP are detected in pure seminoma, it is imperative to acknowledge and manage it as a non‐seminomatous germ cell tumor.^51^, ^100^ Elevated concentrations of HCG in the blood are commonly observed in both seminomas and non‐seminomas. Elevated serum HCG concentrations after orchiectomy serve as an indication of ongoing disease, while the reappearance of HCG levels after chemotherapy‐induced complete remission of metastatic disease highlights the existence of relapse.^51^, ^100^ LDH serves as a marker that lacks specificity, although it possesses an autonomous prognostic significance in males afflicted with advanced TC. Literature has documented that LDH accurately mirrors both the rate of tumor expansion and the extent of tumor mass.^101^ Elevated concentrations of serum lactate dehydrogenase (LDH) have been documented in roughly 80% of advanced seminomas and approximately 60% of non‐seminomas.^101^ While AFP, HCG, and LDH are frequently employed serum markers in the management of TC, their specificity is limited and their detection rate in men with TC is only approximately 60%.^102^ Additionally, the efficacy of these indicators is restricted, and the quantities of these indicators are typically within the “standard” range in approximately 40% of males experiencing disease relapse.^102^ Additionally, the high mobility group proteins HMGA1 and HMGA2 can be classified as nuclear proteins, which exhibit distinct expression patterns depending on the level of differentiation in the testicular germ cell tumor.^103^ The phenomenon of HMGA1/2 being over‐expressed has been documented in pluoripotent embryonal carcinoma, whereas there have been reports of a loss of HMGA1 expression in yolk sac tumor. Furthermore, it has been observed that a loss of HMGA1/2 expression occurs in mature adult tissue of teratoma areas. Consequently, the distinct expression patterns of the HMGA1/2 protein could potentially serve as a valuable tumor marker in TC cases with a challenging histological differential diagnosis.^103^

Placental alkaline phosphatase (PLAP) is regarded as one of the most ancient indicators for GCTs. The ALPP gene, responsible for encoding PLAP, is positioned at chromosome 2q37.1. In normal circumstances, the placenta displays the expression of this enzyme during gestation.^104^ Immunohistochemistry (IHC) reveals the presence of membranous staining in PLAP,^105^ which was initially identified as a highly responsive marker for neoplasia in comparison to the surrounding testicular parenchyma. Specifically, PLAP staining is observed in approximately 99% of germ cell neoplasia in situ (GCNIS), 95%–100% of seminomas, and up to 64% of non‐seminomatous germ cell tumors (NSGCTs), including choriocarcinoma, embryonal carcinoma, and teratomas.^105^ The utility of PLAP is faced with an additional challenge due to its immunoreactivity in nontesticular neoplasms. These neoplasms include gastric adenocarcinomas (22%), adenocarcinoma of the ampulla of Vater (15%), esophageal adenocarcinoma (10%), invasive urothelial carcinoma (4%), cholangiocarcinoma (2%), and adenocarcinoma of the lung (1%).^104^ Many centers in the United States have opted for newer biomarkers, such as SALL4 and OCT3/4, to evaluate postpubertal male GCT in the testis and/or extratesticular sites.^106^, ^107^

Additionally, NANOG and OCT3/4 serve as integral transcription factors essential for the preservation of pluripotency and the perpetuation of self‐renewal in embryonic stem cells. NANOG is observed in the gonocytes located in the maturing testis, as well as in GCNIS, seminomas, and embryonal carcinomas, albeit not in teratomas or YSTs.^108^ NANOG has exhibited exceptional sensitivity (reaching up to 100%) in identifying seminoma and embryonal carcinoma,^109^ thus making it a valuable indicator for these particular histologic subtypes. It has been documented in the literature that OCT3/4 is an additional indicator present in cases of testicular cancer. OCT3/4, belonging to the octamer‐binding protein family, serves as a transcription factor and is recognized as a crucial moderator of pluripotency.^110^ OCT3/4 has been documented as a thoroughly examined indicator for primordial germ cells,^18^ and the expression of this molecule has additionally been documented in carcinoma in situ, seminoma, and embryonal carcinoma.^111^ Although OCT3/4 has the potential to serve as a marker for TC, several reports indicate that this marker is also present in normal adult stem cells and TC which is not derived from germ cells.^112^ Thus, further investigations are required to examine the particularity and accuracy of this indicator.

The zinc‐finger transcription factor, Spalt‐like Protein 4 (SALL4), plays a crucial role in maintaining the pluripotency of embryonic cells. It interacts with other transcription factors associated with pluripotency, including OCT3/4 and NANOG. Moreover, SALL4 exhibits nuclear staining with significant expression levels during embryogenesis and continues to be present in adult germ cells. SALL4 serves as an indicator of high sensitivity for the detection of seminoma (100%), embryonal carcinoma (100%), and Yolk Sac Tumors (93e100%). However, it should be noted that its sensitivity for trophoblastic tumors is comparatively lower. In the realm of germ cell tumors (GCTs), the staining profile about SALL4 is often characterized by a robust and widespread distribution, in stark contrast to the limited and localized staining exhibited by alternate markers such as PLAP.^113^ SALL4 serves as a valuable instrument for discriminating between testicular GCTs and SCSTs, as well as differentiating between metastases or hematologic malignancies in primary tumors, while simultaneously facilitating the identification of GCTs situated in extragonadal sites.^114^, ^115^

Furthermore, SOX proteins have additionally been documented as prospective indicators for TC. SOX2 is an element of the SOX protein lineage and it serves as a transcriptional regulator governing the processes of growth and differentiation.^111^ The expression of SOX2 has been documented in embryonal carcinomas, which represents the undifferentiated component of non‐seminomas. Conversely, it is noteworthy that its presence is not observed in seminomas, yolk sac tumors, and normal spermatogenesis.^111^ In addition, it has also been demonstrated that SOX17 can differentiate carcinoma in situ and seminoma from embryonal carcinoma.^116^ Despite the significant potential of these SOX proteins as novel biomarkers for testicular cancer, their effectiveness as functional diagnostic/prognostic markers still requires empirical validation.

Dieckmann et al.^117^ showed that the levels of MicroRNA‐371a‐3p (M371 Test) in the serum, which is a novel biomarker, could potentially serve as an indicator for the presence of testicular germ cell tumors. The suggestion of utilizing the serum levels of microRNAs (miRs) derived from the miR‐371‐3 and miR‐302/367 clusters as innovative biomarkers for germ cell tumors (GCT) was initially proposed in the year 2011 by Murray et al.^118^ In general, microRNAs (miRs) are a type of small noncoding RNAs that play a role in the epigenetic control of gene expression. Previous research has indicated that miR‐371‐3 and miR‐302/367 exhibit a high level of sensitivity (>90%) and accuracy (>80%) in detecting germ cell tumors (GCTs), with miR‐371a‐3p demonstrating the highest degree of sensitivity and specificity.^119^ The concentrations of this particular miR in the serum appear to have a correlation with both the clinical stage (CS) and the size of the tumor, with levels returning to normal within a time period of less than 24 h after the cancer has been successfully treated.^120^ It is worth mentioning that miR‐371a‐3p expression was detected in seminomas in over 85% of the patient population.^121^

Surveillance of clinical stage I (CSI) testicular germ cell tumors (GCT) is impeded by the limited sensitivity and specificity of existing biomarkers in detecting relapses. Consequently, the examination of microRNA‐371a‐3p serum levels in the DRKS‐00019223 Study offers a potential approach for the identification of recurrence in a follow‐up of stage I testicular germ cell tumors.^120^ This investigation assessed whether serum levels of microRNA371a‐3p (M371 test) possess the capability to: (i) Precisely discern recurrences, (ii) ascertain recurrences earlier than traditional methods, and (iii) potentially forecast relapse by means of heightened postoperative M371 levels.

Through the utilization of proteomic analysis, a collection of nuclear structural proteins has been ascertained that exhibit specificity towards seminomas.^122^ Mass spectrometry and immunoblotting techniques were employed to analyze these proteins. The findings indicated that a protein found in seminoma tissues can be identified as Cell division protein kinase 10 (CDK10).^122^ CDK10 has the potential to be engaged in the process of cellular differentiation and proliferation, consequently making it a potential target for the prognostication of seminomas.^123^ Other proteins that participate in the regulation of the cell cycle have also been employed as prospective prognostic indicators for testicular cancer. As part of an immunohistochemical examination, 19 seminoma and 64 non‐seminoma tissue samples were analyzed, Pestacides et al.^124^ investigated 7 biomarkers that played a role in the regulation of the cell cycle. The researchers communicated that within the markers investigated, p53 and MIB were the sole two markers which exhibited prognostic importance. At threshold levels of 10% and 30% respectively, p53 and MIB‐1 could anticipate the manifestation of progressive disease with an approximate sensitivity of 50%–60% and specificity of 75%–85%.^124^ Additionally, the detection of cell‐free circulating mitochondrial DNA in the blood samples of individuals diagnosed with testicular cancer has emerged as a groundbreaking noninvasive biomarker to monitor disease progression.^125^ Mitochondrial DNA levels were increased in male individuals diagnosed with TC. However, it is worth noting that these levels did not exhibit any significant correlation with various clinic‐pathological variables, encompassing clinical stage, pathological stage and lymph node invasion.^125^ Thus, the continuous exploration of TC biomarkers plays a pivotal role in enhancing disease management.

### 1.9 Treatment of TC

The primary line of therapy for any cancer detected on surgical inspection of a testicular mass is radical inguinal orchiectomy, which includes the removal of the spermatic cord to the internal inguinal ring.^126^, ^127^ A small unilateral or bilateral tumor can be treated with testis‐sparing surgery. If the orchiectomy is life‐threatening, it may be postponed.^126^, ^127^ Therapy following orchiectomy is determined by histology, stage, and prognosis.^126^, ^127^ At the time of diagnosis, half of the germ cell tumors are seminomas and 80%–85% are stage I.^126^, ^127^ Five years following orchiectomy alone, 83%–85% of males with stage I seminoma are free of recurrence; accordingly, surveillance without any further therapy is suggested.^126^, ^127^ Supplemental treatment, mainly etoposide, is used to treat stage II seminomas.^128^ However, non‐seminomas are treated similarly. Stage I nonseminomas had a good prognosis, with 99.1% of disease‐specific 15‐year survivors. It is treatable with active surveillance.^129^ Post‐orchiectomy treatment for stage II and III non‐seminomas includes cisplatin‐dependent chemotherapy and RPLND, depending on lymph node linkage and whether tumor biomarkers stay high after orchiectomy. For stage II nonseminoma, chemotherapy with or without RPLND had a 5‐year relapse‐free rate ranging from 96% to 97%.^130^ If the remaining mass is more than 1 cm, resection is required, however, observation may be required for smaller masses. In general, radiation is not required to treat nonseminomas.^130^ Neoplastic germ cells often lack mature spermatogenesis and have a prominent layer of atypical cells lined along the basement membrane. Intra‐tubular seminoma frequently results in seminoma cells filling seminiferous tubules; this example demonstrates both intratubular and invasive components.^20^

Cisplatin is regarded as a highly potent and greatly esteemed pharmacological agent used in the medical field for combating cancer. Its clinical application extends to treating numerous forms of solid tumors, such as those found in the head and neck, as well as in the testicles and ovaries. However, with the exception of germ cell tumors, the occurrence of cellular resistance, whether inherent or acquired, is frequently encountered and significantly hampers the efficacy of this medication.^121^

Platinum‐based chemotherapy has a remarkable ability to cure GCTs, with cure rates approaching 90% even in patients where metastatic disease is present. However, the biological mechanisms that underlie the remarkable chemocurability of disseminated nonseminomas (NS) are still unknown.^131^ Considering the established data indicating increased SRPK1 expression in the mammalian testicular parenchyma, we identified a possible role for SRPK1 in regulating nonseminomatous GCTs response to platinum‐based chemotherapy.^132^

Surgery in Early Metastatic Seminoma: A Phase II Trial of Retroperitoneal Lymph Node Dissection for Testicular Seminoma With Limited Retroperitoneal Lymphadenopathy as The long‐term toxicities of chemotherapy and radiotherapy can represent a significant burden to testicular cancer survivors. Although there is limited information on its effectiveness in treating early metastatic seminoma, retroperitoneal lymph node dissection (RPLND) is a well‐established treatment with minimal late morbidity for testicular germ cell tumors. A prospective phase II single‐arm, multi‐institutional trial of RPLND is being conducted to evaluate the efficacy of surgery in treating testicular seminoma with clinically low‐volume retroperitoneal lymphadenopathy in early metastatic seminoma cases.^133^

Radiation therapy uses a beam of high‐energy rays (such as gamma rays or x‐rays) or particles (such as electrons, protons, or neutrons) for destruction of malignant cells or inhibit their growth. During TC treatment, radiation is utilized for destruction of malignant cells that metastasize to lymph nodes. Patients with seminoma, which is extremely sensitive to radiation, are typically the ones who receive radiation therapy. Sometimes radiation therapy used following orchiectomy and is directed towards the retroperitoneal lymph nodes for killing any tiny nidus of cancerous cells.

## 2 CONCLUSION

Testicular cancer (TC), accounting for 1% of tumors in males, represents the most widespread neoplasm that observed in young males. With the advent of cisplatin‐based chemotherapy, TC has achieved commendable remission rates, thereby leading to a noteworthy upsurge in the 5‐year survival rate, which currently exceeds 95%. Because individuals with TC are typically diagnosed before reaching the age of 40, it can be anticipated that these males will have a remaining lifespan of approximately 40–50 years subsequent to receiving effective treatment. The potential causes of TC are multifactorial and related to dissimilar pathologies. Accurate diagnosis is essential to detect optimal and appropriate therapy. This can be achieved through blood examinations to detect markers associated with tumors. However, a definitive diagnosis necessitates histopathologic examination of a sample by a medical specialist in pathology. These observations illustrated that TC is multifactorial and has various pathologies, therefore this review aims to revise the prenatal and postnatal causes as well as the therapeutic strategy of TC. Numerous prenatal and postnatal factors are involved in the pathogenesis of TC. However, cryptorchidism is still the major cause of TC. Research efforts are currently underway to detect precise biomarkers of TC that have the potential to enhance the process of diagnosing the disease, monitoring tumor advancement or recurrence, and evaluating the effectiveness of therapeutic interventions. Different biomarkers including AFP, HCG and LDH are used in the diagnosis and follow‐up of patients with TC. However, other biomarkers such as SOX, SOX17 and CDK10 can be applied as biomarkers of TC, but their sensitivity and specificity are conflicting. Therefore, the detection of biomarkers with higher sensitivity and specificity is recommended for both cryptorchidism and TC.

## AUTHOR CONTRIBUTIONS

Kadry M. Sadek, Hazem Y. Abd Ellatief, Sahar F. E. Mahmoud, Athanasios Alexiou: Conceptualization (lead); Visualization; writing – original draft (lead). Marios Papadakis: writing – original draft (lead); funding acquisition (lead). Marwan Al‐Hajeili; Validation, visualization, responding to reviewers comments, writing – review and editing. Hebatallah M. Saad, Gaber El‐Saber Batiha: Conceptualization; supervision; resources (lead); writing – review and editing.

## FUNDING INFORMATION

This work was supported by the University of Witten‐Herdecke Germany.

## CONFLICT OF INTEREST STATEMENT

The authors have stated explicitly that there are no conflicts of interest in connection with this article.

## ETHICS STATEMENT

This review was produced in accordance with the ethics committee of Damanhour University's Faculty of Veterinary Medicine's criteria.

## Data Availability

None.
